# The Synthesis of New Chalcogenides from the System GeTe_6_-Cu and a Layered Structure Based on Them and an Azo Polymer for Application in Optoelectronics

**DOI:** 10.3390/ma18143387

**Published:** 2025-07-18

**Authors:** Yordanka Trifonova, Ani Stoilova, Deyan Dimov, Georgi Mateev, Dimana Nazarova, Lian Nedelchev, Vladislava Ivanova, Vanya Lilova

**Affiliations:** 1 Department of Physics, University of Chemical Technology and Metallurgy, 8 Kl. Ohridski Blvd., 1756 Sofia, Bulgariadimanain@gmail.com (D.N.); lian_n@yahoo.com (L.N.); ivanova_vl@uctm.edu (V.I.);; 2 Institute of Optical Materials and Technologies, Bulgarian Academy of Sciences, Acad. Georgi Bonchev Bl. 109, 1113 Sofia, Bulgaria

**Keywords:** transition metal-doped chalcogenides, azo polymers, Ge-Te-Cu

## Abstract

New bulk chalcogenides from the system (GeTe_6_)_1−x_Cu_x_, where x = 5, 10, 15 and 20 mol%, have been synthesized. The structure and composition of the materials were studied using X-ray powder diffraction (XRD) and energy-dispersive spectroscopy (EDS). Scanning electron microscopy (SEM) was applied to analyze the surface morphology of the samples. Some thermal characteristics such as the glass transition, crystallization and melting temperature and some physico-chemical properties such as the density, compactness and molar and free volumes were also determined. The XRD patterns show sharp diffraction peaks, indicating that the synthesized new bulk materials are crystalline. The following four crystal phases were determined: Te, Cu, CuTe and Cu_2_GeTe_3_. The results from the EDS confirmed the presence of Ge, Te and Cu in the bulk samples in concentrations in good correspondence with those theoretically determined. A layered thin-film material based on Ge_14_Te_81_Cu_5_, which exhibits lower network compactness compared to the other synthesized new chalcogenides, and the azo polymer PAZO was fabricated, and the kinetics of the photoinduced birefringence at 444 nm was measured. The results indicated an increase in the maximal induced birefringence for the layered structure in comparison to the non-doped azo polymer film.

## 1. Introduction

Among the chalcogen family, tellurium-based materials are of special interest as they demonstrate an industrially valuable phase transition when being heated through an electrical current [1] or upon irradiation with light [2], which has already been used in rewriteable optical discs [3] and electronic 3D memory chips [4]. Since the commercialization of GeSbTe [5] and AgInSbTe [6] as information storage media, many investigations have been carried out in order to improve the optical and electrical contrasts between the crystalline and glassy phases, to increase the number of reversible phase transitions, etc. A lot of them involved synthesis of Ge-Sb-Te or quaternary alloy (Ag, In)-doped Sb_2_Te with different concentrations of constituent elements [7,8], addition of dopants [9], introduction of Se and/or S into the matrix [10] or incorporation of another chemical element in place of Sb [11,12]. Regarding the latter, it was reported by Sutou et al. that the crystallization starting time of an amorphous GeCu_2_Te_3_ film is as fast as that of an amorphous GeSbTe film, but it can be re-amorphized by laser irradiation at lower power and with a shorter pulse [13]. Dongol et al. synthesized Ge-Te-Cu bulk glassies with higher thermal stability compared to the Ge-Sb-Te system [14]. Furthermore, it was found that Cu-Ge-Te alloys exhibit a smaller density change upon crystallization and the crystalline phase has a lower optical reflectivity than the amorphous one [15,16]. The observed results have been attributed to the copper d-electrons, which give rise to the different nature of the bonding between the structural units in amorphous and crystalline phases compared to p-bonded chalcogenides [17]. Since then, Ge-Te-based chalcogenides doped with transition metal have received attention as previously overlooked and underexplored materials for optoelectronic application. Many other interesting properties have also been reported for the Ge-Te-Cu system, including resistance to oxidation, relatively high electrical conductivity, low heat conductivity, a high Seebeck coefficient, high transmittance, low absorbance and reflectance in the visible/near-infrared region but high absorbance in the ultraviolet region [18]. In recent years, a significant scientific effort has also been made in the development of composite or hybrid materials combining the advantages of the chalcogenides with those of other materials [19,20,21]. An attractive class among them is represented by azo dyes or polymers, in which the ability of the azochromophores to undergo reversible trans–cis photoisomerization, driven by the presence of the azo (-N=N-) group, induces birefringence. Since then, azobenzene-containing dyes or polymers have been widely investigated as media for the optical recording of information, and, in many studies, they have been doped with nanoparticles, and an enhancement of the maximum induced birefringence in the obtained composites in comparison to pure azo dyes or polymers has been measured [22]. Results on the development of materials combining chalcogenides with azo polymers and their optical performance are purely reported [23,24,25].

In the present article, we report on the synthesis of new chalcogenides from the system Ge-Te-Cu, their structural investigation and the determination of some physico-chemical properties. Furthermore, a layer-by-layer structure based on one of the synthesized new chalcogenides and the azo polymer PAZO is fabricated, and the kinetics of the photoinduced birefringence is studied. The idea for this investigation originates from previously reported by a part of our research group increase in the maximum induced birefringence in PAZO polymer-based composite materials doped with various nanoparticles [26,27,28]. In all these studies, the composite thin-film materials were fabricated through spin coating of a dispersion containing the azo polymer and the particles. In the case which uses chalcogenides as a dopant [25], this technology has shown many challenges related to the proper grinding of the synthesized as bulk material chalcogenides or their insolubility in aqueous media and methanol, in which the PAZO has a good level of solubility. To avoid these drawbacks, in the current work, we prepare a bilayer structure via vacuum thermal evaporation of the chalcogenide and spin coating the PAZO polymer onto it. Tellurium-rich chalcogenides are well known for their high tendency to crystallize, especially in bulk form but also when prepared as a thin film. A replicated crystalline surface structure of the underlying chalcogenide film in the top layer of the PAZO polymer may play a role similar to that of the embedded nanoparticles. To the best of our knowledge, no results from measuring the photoinduced birefringence in a bilayer structure of a chalcogenide from the system Ge-Te-Cu and an azo polymer have been reported up to the present day.

## 2. Materials and Methods

### 2.1. Synthesis of the New Chalcogenides from the Ge-Te-Cu System and Preparation of the Bilayer Structure

The bulk samples from the system (GeTe_6_)_1−x_Cu_x_, where x = 5, 10, 15 and 20 mol%, were synthesized by the conventional melt quenching technique. High-purity Ge, Te and Cu, respectively, Ge, Te-5N and Cu-4N, were weighed in appropriate mol% proportions, put into a quartz ampoule and sealed in a vacuum under a residual pressure of 1.33 × 10^−3^ Pa. The ampoules were then placed in a muffle furnace (Firemagic FM4 Plus, VOP Ltd., Botevgrad, Bulgaria), heated at a rate of 5 × 10^−2^ K/s to a final temperature of 1373 °C and kept at this temperature for 2 h. During the heating process, the ampoules were frequently shaken for better homogenization of the melt constituent. Afterward, the samples were quenched in an ice–water mixture at a cooling rate of 1 × 10^2^ K/s. Finally, the bulk samples were taken from the quartz tube and cut into slices.

**Figure 1:**
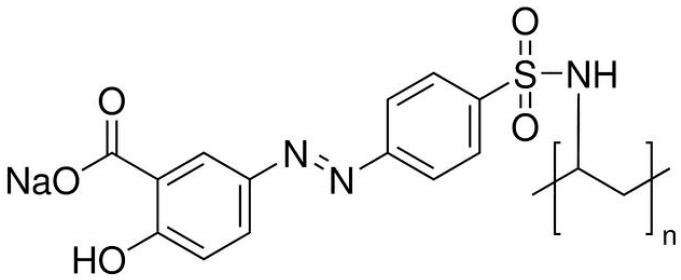
Chemical structure of the PAZO polymer.

The azo polymer poly [1-4-(3-carboxy-4-hydrophenylazo) benzensulfonamido]-1,2-ethanediyl, sodium salt], denoted as PAZO throughout the text, was purchased from Sigma Aldrich (St. Louis, MO, USA, Prod. #346411). It is a polyamide-based azo polymer consisting of two aromatic rings, one para-substituted with a sulfonamide group (–SO_2_NH–) and an azo linkage, and a benzene ring with –OH and –COOH groups (ortho to each other), also attached to the azo linkage. The polyamide backbone is formed by the amine unit of the ethylenediamine group and the sulfonamide group of the azo compound. The structure of the PAZO is shown in Figure 1.

Compared to other azo polymers developed by researchers for optical recording, the PAZO polymer offers the advantages of being easy to access and process. The presence of the sulfonamide group in the PAZO molecule gives it good solubility in water and methanol, enabling simple, cost-effective film fabrication by spin coating or casting. Additionally, the –SO_2_– unit increases the transition dipole moment and lowers the energy barrier for efficient trans–cis photoisomerization. The –NH– unit in the sulfonamide group has the capability to form hydrogen bonds that may act as molecular spacers, maintaining free volume around the azo chromophores by preventing the close packing of polymer chains.

For the preparation of the non-doped PAZO polymer film, 44.6 mg of the PAZO polymer was dissolved in 1200 µL methanol by means of a magnetic stirrer (IKA^®^ RET B 8000, IKA Ltd., Wilmington, DE, USA). The mixture was stirred for one hour at 1700 rpm at room temperature. A drop of 130 µL from the resulting solution was deposited on a quartz substrate and spin coated for 60 s at 1000 rpm. The substrate was cleaned and polished in advance.

For the preparation of the layered structure, the bulk chalcogenide was evaporated from a quartz evaporator on a quartz substrate using the vacuum thermal evaporation technique. The layer was deposited at an evaporation rate of 1 Å/s. The pressure was kept below 2 × 10^−6^ mbar. The thickness and the deposition rate were controlled by a QCM-quartz crystal microbalance (INFICON SQM-160, Inficon Ltd., Bad Ragaz, Switzerland), which, together with the substrate holder, was placed 12 cm above the evaporator. The azo polymer film was deposited onto the chalcogenide film by spin coating a drop of 130 µL from the solution prepared for the fabrication of the non-doped PAZO polymer film for 60 s at 1000 rpm.

### 2.2. Methods Used for Characterization of the Synthesized New Chalcogenides and Measuring the Kinetics of the Photoinduced Birefringence

The XRD experiment was carried out on a “Philips” powder diffractometer working in the Bragg–Brentano (θ–2θ) geometry with CuKα radiation (λ = 154,060 × 10^−12^ m) and a graphite monochromator for the reflected beams. The XRD patterns were obtained for 75 s at a constant scan rate and a reflection angle of 2θ in the scan range from 15° to 70° with a scan step of 0.05°.

A scanning electron microscope (EVO MA 10, Carl Zeiss GmbH, Oberkochen, Germany) connected with EDX (Energy-Dispersive X-ray detector system, Bruker, Billerica, Massachusetts, USA) was used to study the surface morphology (at 10,000× and 30,000× magnification) and the chemical composition of the synthesized new bulk chalcogenides. A micrograph of the Ge_14_Te_81_Cu_5_ film was obtained using a Philips 515 scanning electron microscope (Philips Electron Optics, Eindhoven, Netherlands) with an acceleration voltage of 30 kV and a magnification of 10,000×.

Differential scanning calorimetry (DSC 404 F3, Netzsch, Selb, Germany) was used to determine the glass transition, crystallization and melting temperature of the new chalcogenides. The DSC analysis was carried out by heating 11 mg of each sample in fine powdered form at a constant heating rate of 10 K/min, and the change in heat flow with reference to an empty aluminum pan was measured.

The thicknesses of the films were determined by a high-precision profilometer with an accuracy of ±5 nm. The measured thickness of the pure PAZO polymer film was 300 nm, the chalcogenide film 30 nm and the PAZO polymer spin-coated onto the chalcogenide layer 292 nm.

The density of the bulk samples was estimated by the pycnometer technique with an accuracy of ±0.7% using water as the immersion fluid. The compactness of the materials was calculated according to the equation given below:

(1) $$\delta =\frac{\sum_{i}\frac{c_{i}A_{i}}{\rho_{i}}-\sum_{i}\frac{c_{i}A_{i}}{\rho }}{\sum_{i}\frac{c_{i}A_{i}}{\rho }}$$

where ρ is the density of the sample, and ρ_i_, A_i_ and c_i_ are the density, the atomic mass and the i-th component atomic fraction, respectively.

The molar volume, V_m_, was calculated using Equation (2):

(2) $$V_{m}=\;\frac{1}{\rho }\sum_{i}c_{i}A_{i}$$

The free volume percentage, FVP, was obtained in accordance with Equation (3):

(3) $$FVP=\frac{V_{m}-V_{T}}{V_{m}}\times 100\%$$

where V_T_ is the theoretical molar volume determined as a sum of the molar volumes of the components multiplied by the atomic fraction of each of them (Equation (4)).

(4) $$V_{T}=\sum_{i=1}^{n}c_{i}V_{i}$$

### 2.3. Optical Setup for Measuring the Photoinduced Birefringence

A classical polarimetric setup [29] was used to induce birefringence (Δn) with a pump laser beam (Coherent cube, intensity 330 mW/cm^2^) at a wavelength of 444 nm in a film spot area of approximately 0.2 cm^2^. The writing laser was vertically polarized. A linearly polarized 45° beam from a diode-pumped solid-state (DPSS) laser with a wavelength of 635 nm was used as a probe beam. The Stokes parameters of the output beam were measured by a PAX5710 Polarization Analyzing System (Thorlabs). The value of the induced birefringence was then calculated according to Equation (5):

(5) $$∆n=\frac{\lambda }{2\pi d}arctan\left(\frac{S_{3}}{S_{2}}\right)$$

where λ is the wavelength of the probe laser, d is the film thickness and S_2_ and S_3_ are two of the four components of the Stokes vector.

## 3. Results

**Figure 2:**
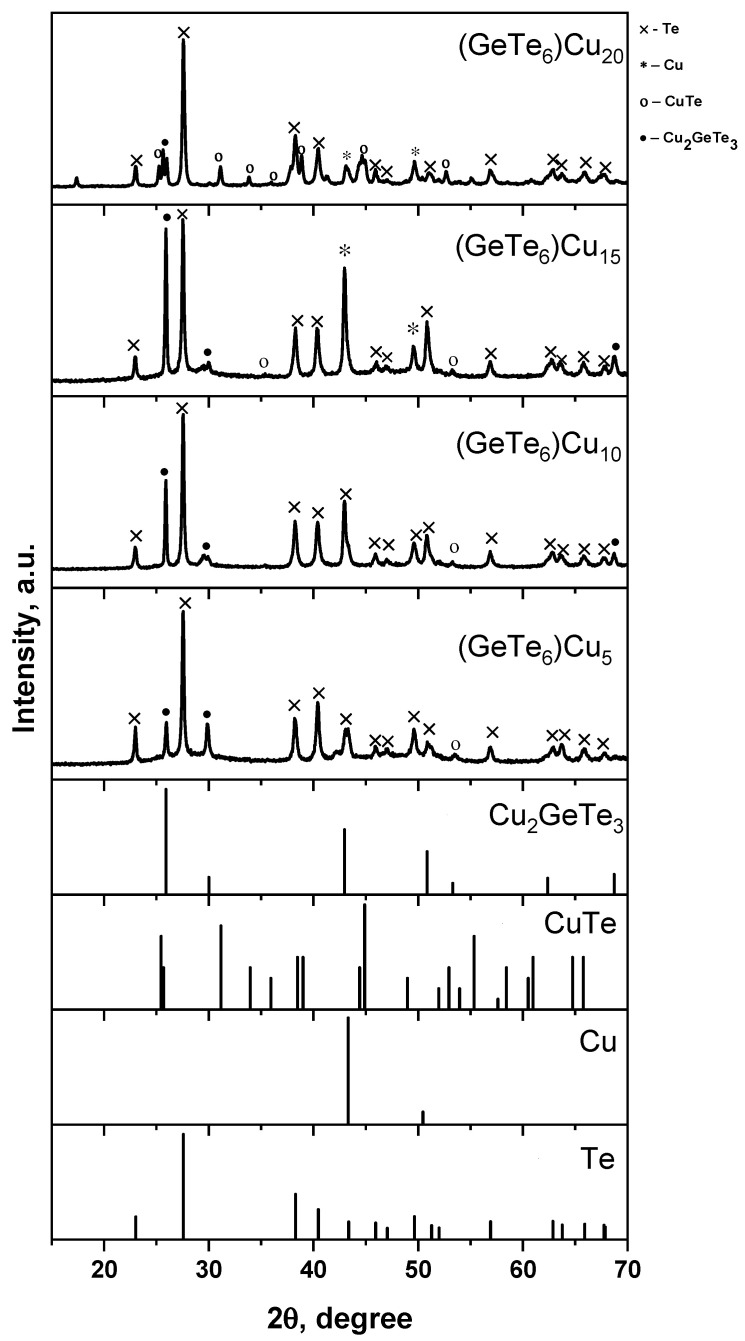
XRD patterns of the samples from the system GeTe_6_-Cu.

The XRD pattern showed sharp diffraction peaks, indicating that the synthesized bulk chalcogenide materials are crystalline (Figure 2). The following four crystal phases were determined: hexagonal Te (JCPDS card no. 36-1452), cubic Cu (JCPDS card no. 85-1326), orthorhombic CuTe (JCPDS card no. 07-0110) and orthorhombic Cu_2_GeTe_3_ [30]. Based on the data obtained, it can be assumed that the synthesized new chalcogenides consist of GeTe_4_ and CuTe_4_ tetrahedra and tellurium chains.

**Figure 3:**
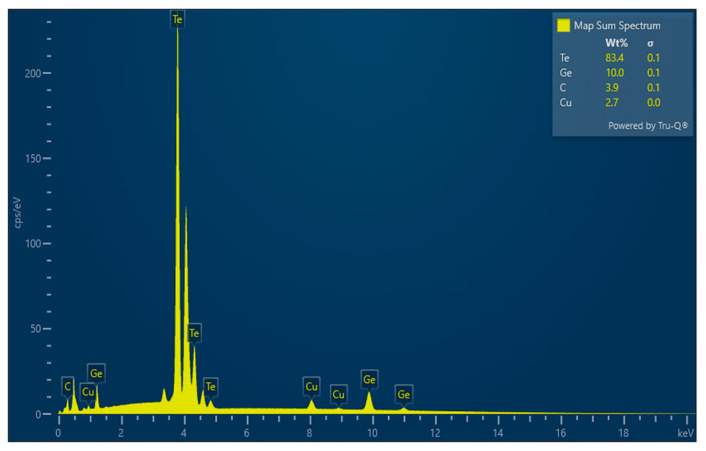
Energy-dispersive spectrum of sample Ge_14_Te_81_Cu_5_.

Figure 3 presents the energy-dispersive spectrum obtained for the sample containing 5 mol% Cu, which indicates the presence of germanium, copper and tellurium in the material studied. Peaks corresponding to these chemical elements were also observed in the spectra of the samples containing 10, 15 and 20 mol% Cu.

Table 1 summarizes the data obtained from the EDS analysis concerning the amount of Ge, Cu and Te present in the samples studied. For comparison, the theoretically determined amount of Ge, Cu and Te for each of the synthesized compositions is given. The results from the EDS confirmed the presence of Ge, Te and Cu in the bulk samples in concentrations in good correspondence with those theoretically determined.

**Figure 4:**
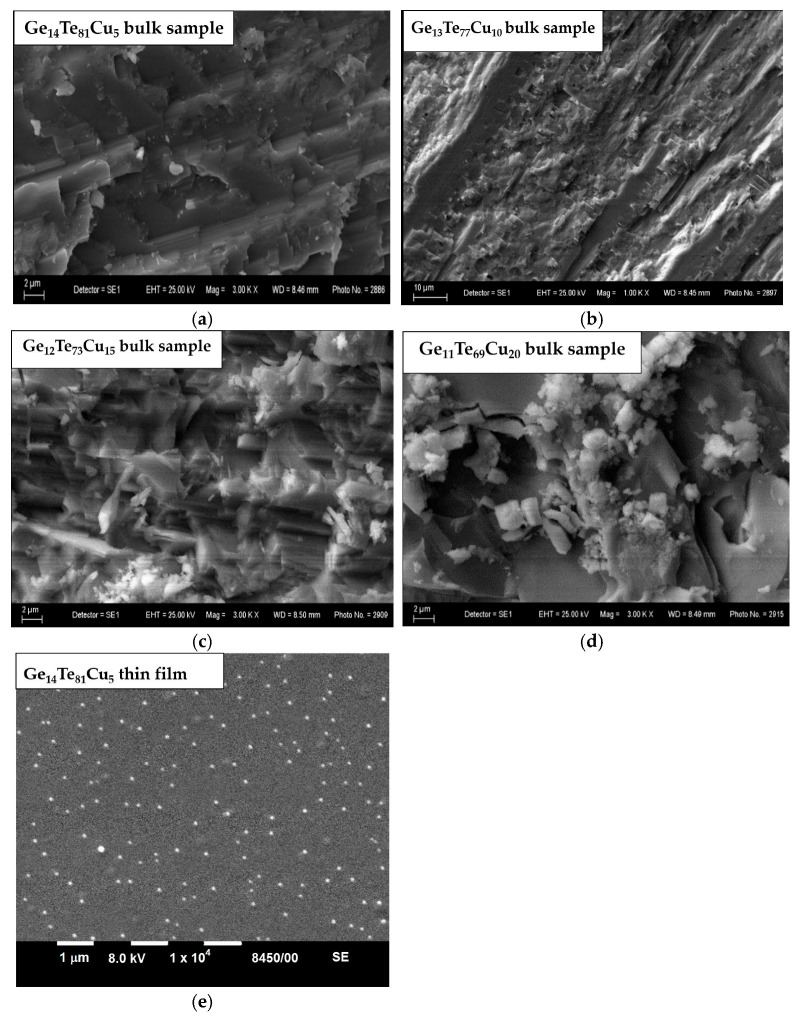
SEM images of the bulk samples studied (**a**–**d**) and the Ge_14_Te_81_Cu_5_ film (**e**).

In Figure 4 are presented SEM images revealing the morphology of the synthesized new bulk materials and the Ge_14_Te_81_Cu_5_ film. The electron micrographs of the bulk sample containing 5 mol% Cu show the presence of single white crystals, different in shape and size. With an increasing Cu amount in the samples, the crystals increase in number and become larger. For the sample doped with 20 mol% Cu, some of the crystals have a rectangular parallelepiped-like shape with a length between 1.0 and 2.2 µm and a height between 1.4 and 2.6 µm. Others form clusters of smaller or larger crystals. The micrograph from the surface of the Ge_14_Te_81_Cu_5_ film shows the presence of a lot of well-defined grains.

**Figure 5:**
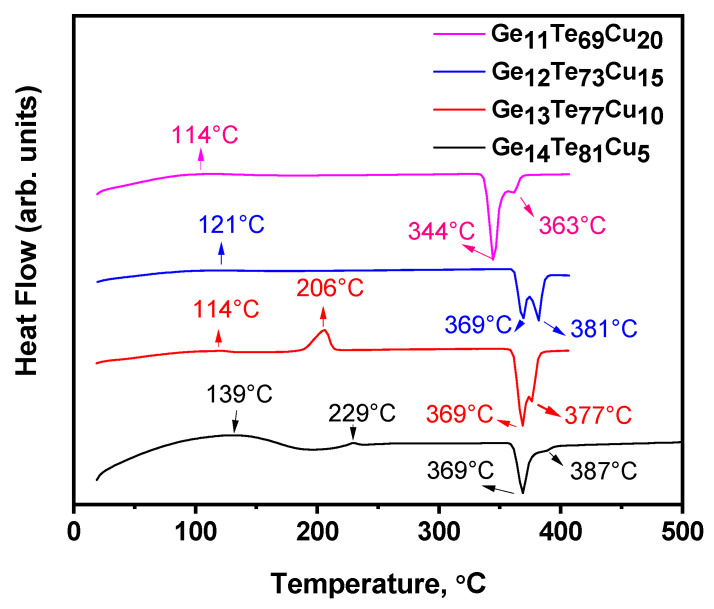
DSC curves of the samples studied.

Figure 5 presents the results from the carried-out thermal analysis. The DSC curve for sample Ge_14_Te_81_Cu_5_ shows a glass transition temperature (Tg) of 139 °C. With increasing Cu content, the Tg decreases and takes a value of Tg = 121 °C for Ge_13_Te_77_Cu_10_ and Tg = 114 °C for Ge_12_Te_73_Cu_15_ and Ge_11_Te_69_Cu_20_. The thermogram of the sample containing 5 mol% Cu shows an exothermic peak at 229 °C, which corresponds to the crystallization temperature (Tc) of the material. For the sample doped with 10 mol% Cu, Tc = 206 °C is more clearly expressed as the observed exothermic peak compared to the one obtained for sample Ge_14_Te_81_Cu_5_. In the thermogram of samples Ge_12_Te_73_Cu_15_ and Ge_11_Te_69_Cu_20_, such a peak is not observed. The appearance of the exothermic peak at 206 °C for the sample doped with 5 mol% indicates that the material crystallizes during heating as well. The DSC curve for sample Ge_14_Te_81_Cu_5_ shows a clearly expressed endothermic peak corresponding to a melting temperature (Tm) of 369 °C. The shoulder at 387 °C indicates the presence of two crystalline phases, the first one being at a higher concentration. Based on the obtained XRD data, it could be assumed that the peak at 369 °C corresponds to the tellurium and the shoulder at 387 °C to the Cu_2_GeTe_3_ crystalline phase. The DSC curves for the samples containing 10 mol% and 15 mol% Cu show a double melting peak, with two clearly expressed maxima at 369 °C and 381 °C for Ge_13_Te_77_Cu_10_ and at 369 °C and 377 °C for Ge_12_Te_73_Cu_15_, respectively, which could be associated with the presence of tellurium and Cu_2_GeTe_3_ crystalline phases in the material. The thermogram of sample Ge_11_Te_69_Cu_20_ shows an endothermic peak corresponding to a melting temperature of 344 °C and a shoulder at 363 °C indicating the presence of two crystalline phases. Based on the obtained XRD data, we assume that these phases are Te and CuTe.

**Figure 6:**
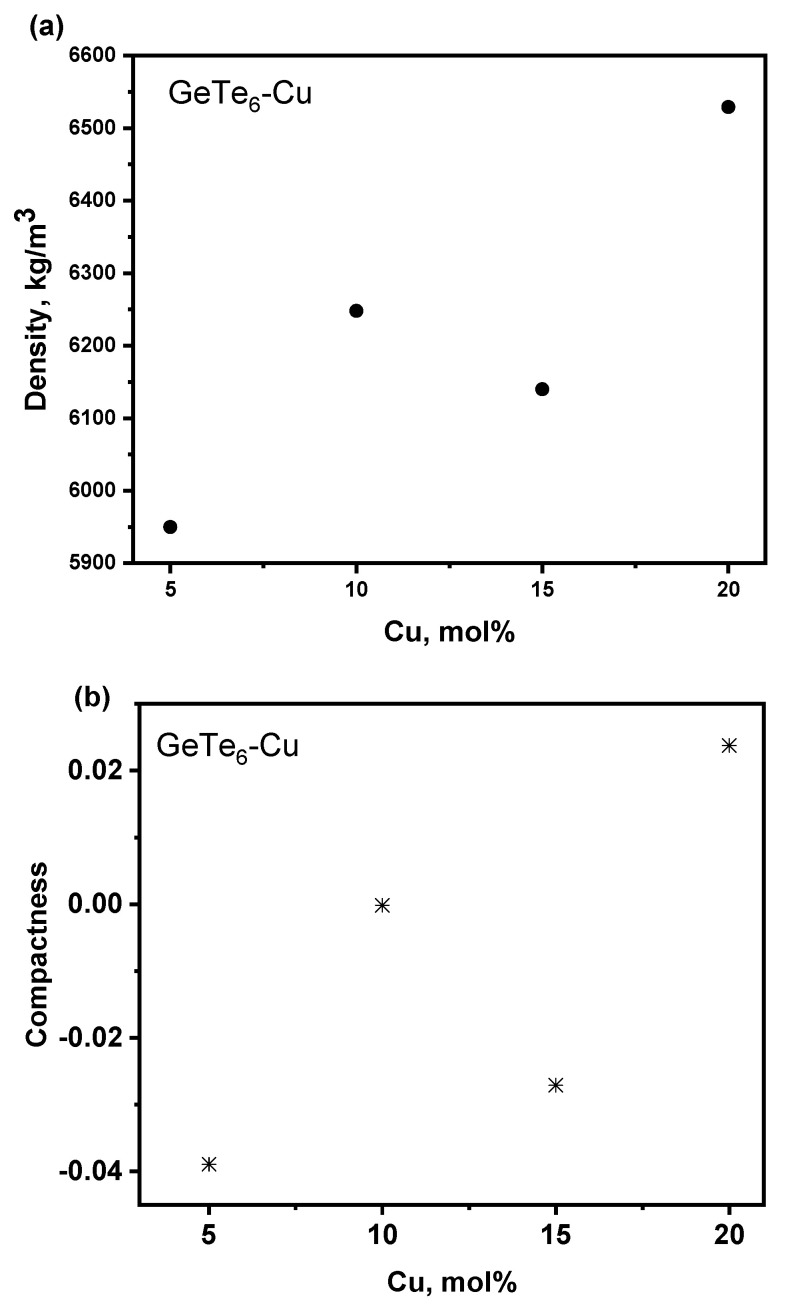

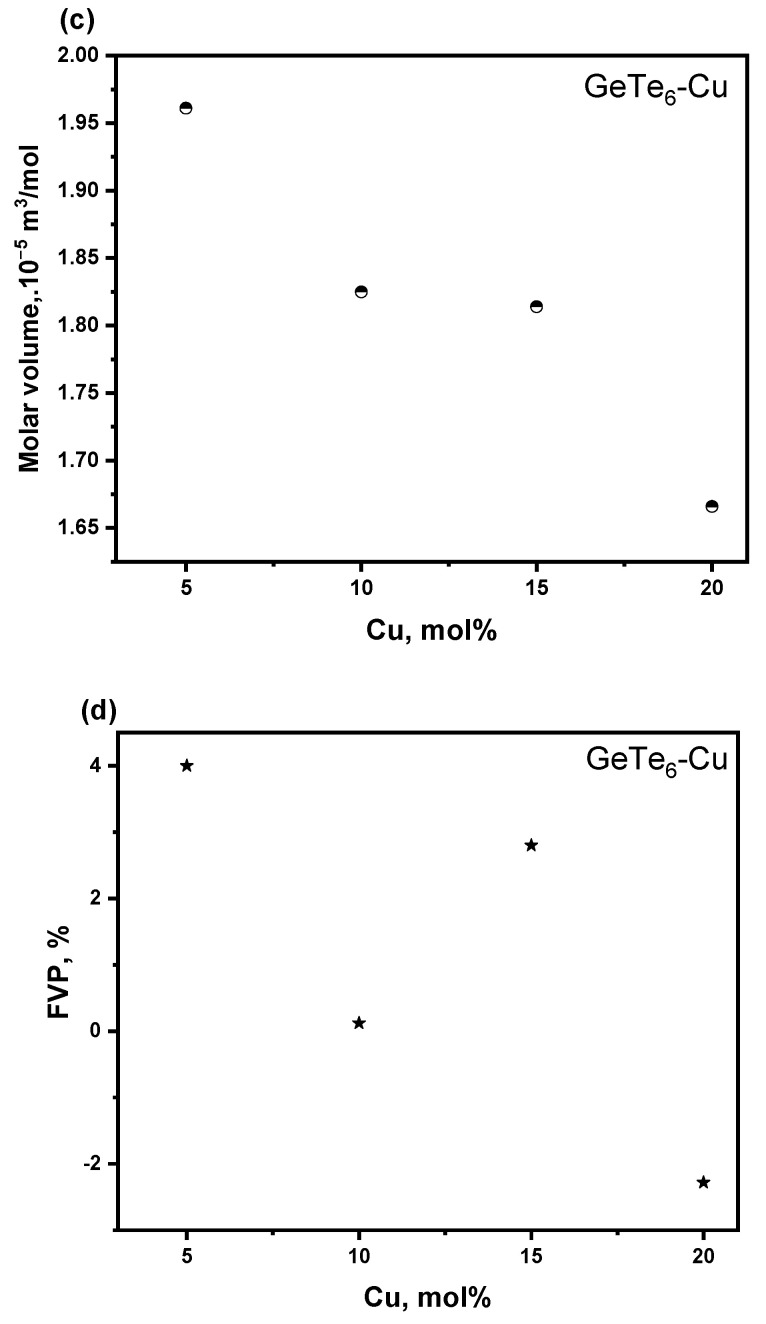
Density (**a**), compactness (**b**), molar volume (**c**) and FVP (**d**) dependence on the composition.

In Table 2 are presented the obtained values for the density, compactness, molar volume and free volume percentage of all the samples studied, and their dependence on the copper concentration is shown in Figure 6. As can be seen, the value of the density increases as the copper content increases. Since the size of the Ge and Gu atoms is similar (atomic radii of 1.22 and 1.28 Å, respectively), the observed increase in the density is probably due to the lowering in the tellurium content, as the tellurium atoms possess bigger atomic radii of 1.40 Å. The introduction of smaller atoms leads to the formation of a more dense structure, as confirmed by other studies [31]. The dependence of the compactness on the copper concentration follows the same trend as the density: an increase with increasing Cu content is observed. The increase in the concentration of atoms smaller in atomic radius and less heavy in weight (the atomic weight of Ge, Te and Cu is, respectively, 72.6 g·mol^−1^, 127.7 g·mol^−1^ and 63.5 g·mol^−1^) leads to the formation of a more compact and dense packaged structure. Figure 6c presents the molar volume of the materials as a function of the composition, and it can be seen that V_m_ decreases with the addition of Cu. The observed reduction in the values can be viewed as defined by the following two aspects: a decrease in the molecular weight and an increase in the density of the material. From Figure 6d, it can be seen that the free volume percentage has the lowest value for the sample containing 20 mol% copper and the highest value for the sample doped with 5 mol% Cu.

**Figure 7:**
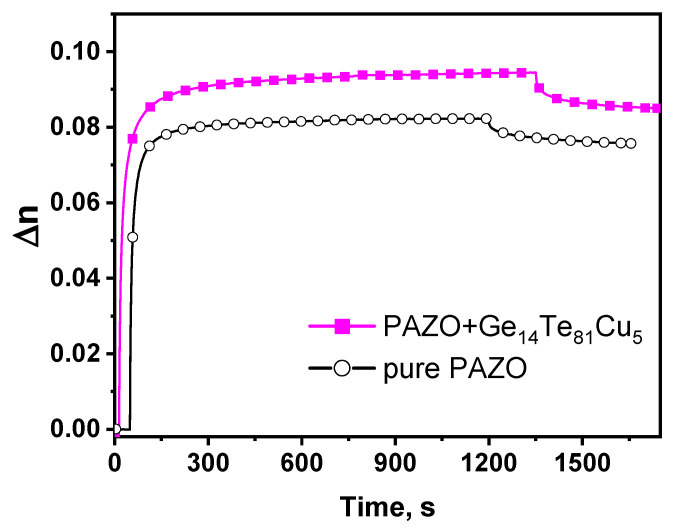
Birefringence kinetics for the samples studied induced at 444 nm.

In Figure 7 are presented the results from measuring the kinetics of the birefringence induced in the samples studied with a pump laser at a 444 nm recording wavelength.

For the pure PAZO polymer film, the maximal induced birefringence has a value of Δn_max_ = 0.083. For the layer-by-layer structure, an increase in the maximal induced birefringence, Δn_max_ = 0.095, was measured. From the kinetic curves, two important characteristics of the photoinduced birefringence have been determined—the time response (τ), or the time needed to reach 80% of Δn_max_, and the time stability (r_t_), or the ratio of the anisotropy retained after switching off the recording laser to the maximal value of the photoinduced anisotropy during the recording. The results are summarized in Table 3.

The faster kinetics of the pure PAZO polymer film compared to the bilayer structure could be attributed to the absorption of writing light by the chalcogenide, which reduces the amount of effective light reaching the PAZO chromophores. After switching off the pump beam, both samples show less than 10% birefringence decay within 300 s.

## 4. Discussion

Generally, the observed higher value of Δn_max_ for the bilayer structure based on the azo polymer and Ge_14_Te_81_Cu_5_ in comparison to that of the pure PAZO film could be viewed as resulting from changes in the free volume and/or the increased number of scattering and reflectance points within the top layer due to the underlying film morphology (i); and interference effects between light reflected at the boundaries between the two layers (ii). Considering (i), the polymer layer spin coated onto the chalcogenide film might closely replicate the surface texture of the underlying film [32,33]. As shown in Figure 4e, there are a lot of grains on the surface of the chalcogenide film, which, when “copied” into the structure of the azo polymer layer, increase the path length of the light within the film and provide opportunity for interaction of the photons with the azo chromophores at various angles. In such cases, the Ge_14_Te_81_Cu_5_ film plays a role similar to that of the nanoparticles used as a dopant in PAZO polymer-based composite materials, which, according to the main reported up-to-date hypothesis, consists of increasing the free volume around the azo chromophores, making the number of molecules capable of isomerization higher, or increasing the scattering inside the composite, allowing excitation and reorientation of originally off-plane chromophores that would otherwise not contribute to the birefringence [22]. Considering (ii), when light interacts with a layer-by-layer structure, reflections or scatterings from the interface between the two layers can undergo constructive interference, resulting in increased light intensity and then in enhanced birefringence of the top layer. Furthermore, scattering and reflectance effects may alter the polarization stay of the incident light. These changes can lead to the activation of azo chromophores, even those that are not inherently sensitive to the polarization of the incident light. These assumptions are under consideration in our ongoing investigations.

## 5. Conclusions

New chalcogenides from the system (GeTe_6_)_1−x_Cu_x_, where x = 5, 10, 15 and 20 mol%, have successfully been synthesized by one-step melt quenching technique. The carried-out XRD analysis shows the presence of Te, CuTe and Cu_2_GeTe_3_ phases in the samples doped with 5 mol% and 10 mol% Cu and of Te, CuTe, Cu_2_GeTe_3_ and Cu phases in the samples doped with 15 mol% and 20 mol% copper. The DSC measurement revealed that, with increasing copper content, the glass transition temperature decreases from 139 °C to 114 °C while the crystallization temperature follows the same trend. According to the calculated values of the compactness and free volume percentage of the synthesized new bulk chalcogenides, the most flexible structure is possessed by sample Ge_14_Te_81_Cu_5_. A layered thin-film material based on the synthesized new Ge_14_Te_81_Cu_5_ chalcogenide and the azo polymer PAZO has been fabricated, and the kinetics of the photoinduced birefringence at 444 nm has been measured. The results indicate a 14.46% increase in the maximal induced birefringence for the layered structure (Δn_max_ = 0.095) in comparison to the non-doped azo polymer film (Δn_max_ = 0.083).

## Figures and Tables

**Table 1:** EDS elemental analysis (expressed in percentage of weight) and comparison with the calculated elemental concentration in mol% converted to weight percent (wt.%).

Sample mol%	Theoretically Determined Elemental Concentration, wt.%	Elemental Concentration Determined by EDS, wt.%
Ge_14_Te_81_Cu_5_	Ge—8.7 Te—88.6 Cu—2.7	Ge—10.0 ± 0.1 Te—83.4 ± 0.1 Cu—2.7 ± 0.0
Ge_13_Te_77_Cu_10_	Ge—8.3 Te—86.1 Cu—5.6	Ge—6.6 ± 0.1 Te—82.2 ± 0.1 Cu—5.6 ± 0.0
Ge_12_Te_73_Cu_15_	Ge—7.8 Te—83.6 Cu—8.6	Ge—7.9 ± 0.1 Te—76.5 ± 0.1 Cu—9.7 ± 0.0
Ge_11_Te_69_Cu_20_	Ge—7.3 Te—81.0 Cu—11.7	Ge—0.7 ± 0.0 Te—71.3 ± 0.1 Cu—9.2 ± 0.0

**Table 2:** Physico-chemical properties of the bulk samples studied.

Sample	Density, 10^3^ kg/m^3^	Compactness, 10^−2^	Molar Volume, 10^−5^ m^3^/mol	FVP, %
Ge_14_Te_81_Cu_5_	5.950	−3.890	1.961	4.00
Ge_13_Te_77_Cu_10_	6.248	−0.015	1.825	0.12
Ge_12_Te_73_Cu_15_	6.140	−2.709	1.814	2.80
Ge_11_Te_69_Cu_20_	6.529	2.378	1.666	−2.28

**Table 3:** The time response (τ) and time stability (r_300_) of the induced birefringence.

Sample	τ, [s]	r_300_, [%]
Pure PAZO film	25	92.7
Bilayer structure	38	90.4

## Data Availability

The original contributions presented in this study are included in the article. Further inquiries can be directed to the corresponding author.
